# Compulsory Citizenship Behavior and Employee Creativity: Creative Self-Efficacy as a Mediator and Negative Affect as a Moderator

**DOI:** 10.3389/fpsyg.2020.01640

**Published:** 2020-07-21

**Authors:** Peixu He, Qiongyao Zhou, Hongdan Zhao, Cuiling Jiang, Yenchun Jim Wu

**Affiliations:** ^1^Research Center of Business Management & Oriental Enterprise Management Research Center, Business School, Huaqiao University, Quanzhou, China; ^2^School of Management, Shanghai University, Shanghai, China; ^3^Department of Management, Kedge Business School, Talence, France; ^4^Graduate Institute of Global Business and Strategy, National Taiwan Normal University, Taipei, Taiwan

**Keywords:** organizational citizenship behavior, compulsory citizenship behavior, employee creativity, creative self-efficacy, negative affect

## Abstract

Workplace stressors were identified to have critical impacts on employee creativity. However, little is known about how and when involuntary citizenship behavior [i.e., compulsory citizenship behavior (CCB)]-induced stress might exert an influence on employee creativity. To fill this knowledge gap, the present study firstly develops a moderated mediation model to investigate the CCB–employee creativity association as well as the underlying mechanism and contextual condition of this relationship. By integrating social cognitive theory such as self-efficacy theory and conservation of resources (COR) theory, we propose that CCB predicts employee creativity through the mediating role of creative self-efficacy (CSE), with the individual characteristics (i.e., personality traits) of negative affect acting as a boundary condition. Using two-wave time-lagged survey data collected from a sample of 251 frontline employees in 10 manufacturing firms in Southern China, the results show that: (a) CSE mediates the negative relationship between CCB and employee creativity; (b) negative affect moderates the relationship between CCB and CSE; (c) negative affect moderates the indirect influence of CCB on employee creativity through CSE. As the level of negative affect rises, this indirect relationship is stronger. Finally, important theoretical and managerial implications and promising avenues for future research are addressed.

## Introduction

Employee creativity refers to the generation of novel and useful ideas (Amabile, 1983). With the ongoing business competition and uncertain global economic environment, innovation has been widely used to enhance service delivery (Biljohn and Lues, 2019), and employee creativity has been widely recognized to be essential for organizational innovation, sustainability, and long-term performance (George and Zhou, 2001; Antwi et al., 2019; Baradarani and Kilic, 2018; Hermida et al., 2019). Accordingly, researchers have focused on identifying the antecedents that affect employee creativity, such as abusive supervision (Aryee et al., 2007), work autonomy (Zhang et al., 2017), and work stressors (Noefer et al., 2009). Among them, work stressors are frequently mentioned in the organizational behavior (OB) research because they are recognized as quite prevalent but invisible conditions in the organizations related to management strategies.

However, conclusions regarding the influence of work stressors on creativity from previous literature had contradictory results: positive, negative, curvilinear, or no significant effects (Byron et al., 2010). One possible reason for the inconclusive results might be that previous studies have neglected the nature of stressors. The challenge–hindrance stressors framework suggests that different stressors will affect employee creativity in different ways. Indeed, hindrance stressors (such as organizational politics, bureaucratic habits, role conflicts and ambiguity, job insecurity) (Cavanaugh et al., 2000) have been consistently identified to have negative relationships with creativity (Zhou, 2003; Byron et al., 2010; Geng et al., 2014; Montani et al., 2017). For instance, drawing on cognitive appraisal theory of stress and coping, Naseer et al. (2020) have determined that hindrance stressors can undermine key employee performance outcomes such as creativity. However, in relation to specific hindrance pressures, the prior studies lack in-depth and detailed microscopic research findings (Probst et al., 2007). Therefore, recent research has questioned whether different kinds of hindrance stressors have the same degrees of influence and calls for a more detailed view of hindrance stressors by focusing on the influence of non-traditional and unconventional workplace stressors to employ creativity (Janssen et al., 2004; Byron et al., 2010).

In order to shed light on these insufficient findings, we choose compulsory citizenship behavior (CCB) as a kind of specific hindrance pressure in our study. Vigoda-Gadot (2006) argued that CCB is “a unique segment of citizenship behavior or extra-role behavior, one that is less voluntary but still expresses extra effort at work” (p. 81). Indeed, CCB is quite prevalent in the workplace. As a non-traditional and unconventional stressor, the influences of CCB are expected to be negative and even harmful to employees’ well-being and organizations’ further development (Zhao et al., 2013). Employees who interpret citizenship pressure negatively are likely to engage less in taking individual initiatives and which have negative impact on performance (Bolino and Turnley, 2005). Particularly, He et al. (2019a) have highlighted the positive influence of CCB on counterproductive work behaviors (CWBs). Spanouli and Hofmans (2016) research also showed that elicited organizational citizenship behavior [(OCB) i.e., increased citizenship demands] can have an adverse effect for the organization by backfiring and leading to increased CWBs. Although potential personal or organizational costs of performing CCBs have been raised in prior studies, little is known about the relationship between CCBs and employee creativity. Therefore, drawing upon the abovementioned findings, we tend to focus explicitly on the outcome of CCB to employee creativity. On the one hand, employees’ dissatisfaction with their work induced by CCBs will prevent them from engaging in developing new ideas. Because of those compulsory extra-role requirements against employees’ will (Vigoda-Gadot, 2007), the individual has to invest time and psychological resources on private affairs to cope with them, thereby the motives and engagement in creativity will be decreased, and the mental exhaustion can dilute their internal creative motivation (Hobfoll, 2001). On the other hand, prior research has confirmed that work–life balance can promote employees’ productivity and motivation (Lazar et al., 2010; Fotiadis et al., 2019). However, CCBs are likely to destroy employees’ work–life balance and trigger work–life conflicts, which generally increase employees’ psychological stress and decrease their psychological well-being (Hofmann and Stokburger-Sauer, 2017). This could ultimately lead to a lower level of employee feelings of self-efficacy and employee enthusiasm in behaving creatively in the organizations, as well as a higher level of work-withdrawal behaviors.

Logically, our following objective is to examine the potential processes responsible for the CCB–employee creativity relationship. Many findings have clarified the importance to pay attention to the importance of cognitive and motivated perspectives for the better understanding of the workplace stressors–employee creativity relationship (e.g., Fida et al., 2015; Tang, 2019; Tu et al., 2019). Utilizing a self-efficacy theory (Tierney and Farmer, 2002), our study discusses the mediating mechanism of CCB–employee creativity relationship by examining the influence of employees’ creative self-efficacy (CSE), which is a particular type of self-efficacy and creativity-focused motivational attribute, referring to an employee’s belief that he/she has the ability to perform successfully and produce creative outcomes (Tierney and Farmer, 2002). As a domain-specific form of efficacy, CSE is considered to be a crucial requirement in achieving creative performance (Choi, 2004; Egan, 2005), when employees are confident in their abilities in some specific fields, they are likely to generate creative ideas and accomplish creative outcomes. Meanwhile, Chong and Ma (2010) mentioned that individuals were unable to perform creatively if they had no confidence in their creative ability. CCB makes employees frequently face strong social or managerial pressures and destroys the formal and free atmosphere, which will affect the perceptions of their creative ability; eventually, employee creativity can be influenced. Therefore, CCB can decrease employee creativity because it hinders employees’ CSE.

Despite some useful and insightful conclusions of this past literature, examining the boundary conditions of the CCB–employees’ CSE relationship remains underexplored. CCB forces employees to finish tasks, making the focal employees feel stressed and threatened, which will decrease employees’ intrinsic motivation of creative thinking. However, different personalities (such as negative affect) will react differently to CCB. Negative affect refers to “a general dimension of subjective distress and unpleasant engagement that includes a variety of aversive mood states, including anger, contempt, disgust, guilt, fear, and nervousness” (Watson et al., 1988, p. 1063). According to the conservation of resources (COR) theory, by satisfying the basic psychological needs, job resources can foster extrinsic motivation of employees (Van den Broeck et al., 2008). As an important internal resource, affect/emotions could influence employees’ perceptions on resource loss. When employees have positive affect/emotions, it means that they may have rich internal resources, which could buffer the resource loss feeling caused by the CCB. On the contrary, when employees have negative affect/emotions, this means that they possess poor internal resources. Under this condition, the consumption of employee resources requested by CCB is likely to deepen their feeling of resource loss. For employees with a higher level of negative affect, CCB will cause the employee to respond with more narrow, anxious, and problem-based responses. Because when employees are forced to behave extra-role behaviors against their will, they are not likely to invest resources in exchange for positive emotions or outcomes. Instead, they will tend to conserve the available resources, which in turn reinforce the negative relationship between CCB and employees’ CSE. From another perspective, according to broaden-and-build theory of positive emotions, positive affect/emotions would broaden people’s cognition and attention, while negative affect/emotions would limit individuals’ cognitive ability and creative thinking (Fredrickson, 2001). Song et al. (2019) have conducted a research on generation Y in China. The findings show that negative affect could lead to employees’ unwillingness and “cannot do” for innovative behaviors through the motivation and cognition path. With negative affect, Generation Y employees are less likely to have creativity engagement at work, but adopting the work attitude and behaviors that are consistent with their negative affect. This would lead to employees’ reduced creativity performance, divergent thinking and actions. Based on the above, we conclude that the negative effect might play a regulating role between CCB and employees’ CSE.

**FIGURE 1:**
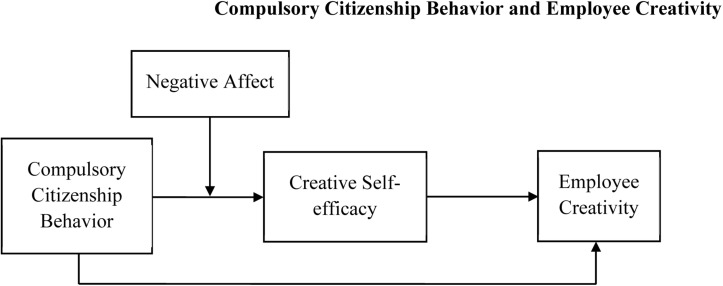
Hypothesized moderated mediation model of processes linking CCB and employee creativity. CCB, compulsory citizenship behavior.

In sum, this study aims to make some theoretical and practical contributions to both the CCB and creativity literature by offering a conceptual model that links CCB, employees’ CSE, employee creativity, and negative affect. First, OB researchers and practitioners have long focused on the positive consequences of OCB. However, the nature and the dark side of enforced/involuntary citizenship behaviors have not been fully discussed in the prior literature so far. In an effort to enhance our understanding of CCB, the present study examines its consequences. Our findings could not only enrich the existing research on the result variables of CCB but also provide a theoretical and empirical support for explaining the negative impact of CCB. Second, prior studies mainly examine the positive impacts of OCB on employees’ creativity, but ignoring the respective negative impacts. Although some scholars start to point out that CCB can have a negative effect on employee creativity (e.g., Vigoda-Gadot, 2007), there is still a significant lack of related empirical studies. Based on the self-efficacy theory, this study bridges the gap by testing the relationship between CCB and employee creativity, as well as the mediating role of employees’ CSE between CCB and employee creativity. Our study refines and updates the application of social cognitive theory in more areas, providing a theoretical framework that helps better understand the mechanism of CCB’s effect on employees’ creativity and performance. Third, this study explores the regulating role of negative affect in affecting employee creativity based on the COR theory, which sheds new light on the theoretical knowledge of how CCB can reduce employees’ CSE and employee creativity. Our findings also provide empirical support for the emotion-cognitive interaction model. We advance the theoretical understanding of the influencing mechanism of affective personality traits on individual cognition and behavior under stress. Last, prior studies have examined the impacts of citizenship pressure and CCB on employees’ negative psychology (e.g., exhaustion/burnout and moral disengagement) and negative workplace behaviors (e.g., deviance/CWB and unethical workplace behaviors) but have ignored their impacts on employees’ positive psychology (e.g., self-efficacy) and positive workplace behaviors (e.g., innovative work behavior). This study simultaneously investigated the roles of positive (employee self-efficacy and creativity) and negative (CCB and negative affect) organizational psychology and behavior and provided explanation to how they integrate with each other. Figure 1 is the theoretical model of this study.

## Theoretical Background and Hypotheses Development

### Compulsory Citizenship Behavior and Employee Creativity

Prior research on OCB—“individual behavior that is discretionary, not directly or explicitly recognized by the formal reward system, and that in aggregate promotes the effective functioning of the organization” (Organ, 1988, p. 4)—has advocated its positive influences on employees and organizations. However, in recent years, there is an emerging stream of research starts to question the exclusive discretionary nature of OCB (Youn et al., 2017; Germeys et al., 2019) and examine the potential negative consequences of OCB (Deery et al., 2017; De Clercq et al., 2019; He et al., 2019a). In particular, the destructive effects of the enforced/non-voluntary version of citizenship behavior, which was described by Vigoda-Gadot (2006) as CCB, have attracted considerable attention from OB and human resource management (HRM) scholars (e.g., Zhao et al., 2014; He et al., 2018, 2019a). CCB has been seen as a kind of hindrance stressor in the workplace (He et al., 2019a). Because, as a unique form of extra-role behavior that reflects “the exploitative and abusive tendency of supervisors and managements” (Vigoda-Gadot, 2007, p. 377), CCB often triggers role conflict and ambiguity and even leads to employees’ perception of organizational injustice and job insecurity (Zhao et al., 2014; Zhang L. et al., 2018). When expending extra efforts at work (e.g., engaging in helping behaviors toward the coworkers and supervisors beyond the formal job obligations) due to facing strong social or managerial pressures in the workplace, employees are likely to feel role overload, burnout, and anxiety. Citizenship pressure is extremely harmful to employees’ work motivation (De Clercq et al., 2019) and can seriously endanger their health and well-being (Deery et al., 2017; He et al., 2019a; Somech and Bogler, 2019) as it might be positively associated with job stress/burnout (Zhang L. et al., 2018), work–family/work–leisure conflict and intention to quit (Bolino and Turnley, 2005; Bolino et al., 2010; Youn et al., 2017; Banwo and Du, 2020). For instance, a significant relationship between nurses’ CCB and their experienced job stress was found by Ünaldi Baydin et al. (2020). In addition, Germeys et al. (2019) argued that experiencing citizenship pressure will diminish employees’ autonomously/spontaneously motivated OCB while increasing their controlled OCB. They also successfully linked citizenship pressure to work–home conflict by evoking the performance of controlled OCB.

Being considered as work demands, recent research has indicated that hindrance stressors (including role conflict, role ambiguity, job insecurity, organizational politics, red tape, and hassles) can constrain personal growth and hamper an individual’s ability to achieve work-related accomplishment or personal goals (Cavanaugh et al., 2000; LePine et al., 2005; Crawford et al., 2010) and can impede employee creativity (Hon et al., 2013; De Spiegelaere et al., 2014; Ren and Zhang, 2015; Zhang et al., 2016; Du et al., 2019; Naseer et al., 2020). Specifically, engaging in creative activities is often accompanied by uncertainties and challenges and thus generally requires more professional skills, enduring extra effort, time, and energy and taking on additional risk of innovation failure. However, hindrance stressors continuously consume employees’ limited and invaluable resources (Cavanaugh et al., 2000). The COR theory provides scholars with a lens for investigating the generating process of stress and the strategies that individuals adopt to cope with stress and retain and protect resources that have instrumental/symbolic value (Hobfoll, 1989). According to the COR theory, people fundamentally strive for resource surplus while avoiding resources loss (Xu et al., 2015). Hobfoll (1989, 2001) suggested that chronic stressors endow employees with intense motivation and willingness to conserve their resources and protect them from potential further resource loss and depletion. In addition, LePine et al. (2005) indicated that employees under hindrance stressors cannot get sufficient rewards from their extra efforts on satisfying the stressful demands. Therefore, it is not difficult to speculate that, when confronted with sustained hindrance stressors, employees will tend to “spend less effort on creative activities to protect their resources” (Du et al., 2019, p. 7). Supporting this reasoning and referring to cognitive resource theory, Vecchio (1990) argues that high stress will affect individuals’ cognitive resources and damage individuals’ ability to think creatively and rationally.

Citizenship behaviors are not explicitly recognized by an organization’s formal reward system (Organ, 1988). Consequently, excessive organizational pressures for citizenship behaviors can lead to citizenship fatigue—a state in which employees feel worn out, tired, or on edge attributed to engaging in OCBs (Bolino et al., 2015; De Clercq et al., 2019). As for CCB, it stands as a salient social pressure in the organization (Vigoda-Gadot, 2007) that threatens and depletes employees’ desirable resources. When employees are expected to invest more efforts in their jobs and they are forced to engage in extra-role work activities without any formal rewards, they unavoidably need to spend much more time and energy to struggle and survive. As a result, CCB generates employees’ physical fatigue, emotional exhaustion, and cognitive strain, which ultimately undermine their work life quality and work–family balance. For example, based on an in-depth interview, Somech and Bogler (2019) suggested that citizenship pressure endows teachers a feeling of depletion of physical and mental resources. They will feel frustrated and angry when they find they do not have the ability to comply with the organization’s demands. From the perspective of the COR theory, employees under CCBs know that their efforts will not get rewarded, which enhances their powerless feeling of work, leads them to perform withdrawal behavior (i.e., distancing themselves from their jobs or avoiding job-related tasks) (Somech and Bogler, 2019), and keeps them from creative process engagement. Furthermore, prior research has pointed out that creative ideas are generated only when employees have a feeling of freedom from control and of safety (Byron et al., 2010). However, the controlled motivation-driven citizenship behaviors (i.e., citizenship behaviors that are driven by outside forces) will undoubtedly lead to a sense of strong pressure and insecurity and thereby inhibit the generation of creative ideas. In other words, CCB reduces the employees’ autonomy at work, which is often an important condition for employees to exert their creativity (Song et al., 2019). At last, Tepper (2007) pointed out that subordinates often try to engage in passive or avoidant behaviors to alleviate the physical and psychological discomforts caused by workplace stressors. In order to avoid further resource depletion arising from further “be forced” encounters (i.e., CCBs), employees would be more likely to isolate themselves from CCBs and remain silent by turning a blind eye to crucial information and problems and reserving opinions or withholding suggestions (He et al., 2018, 2019a). These silence or knowledge-hiding behaviors will, in turn, harm employees’ creativity (Černe et al., 2014; Bogilović et al., 2017). In sum, when facing increased CCBs, employees will tend to conserve their remaining resources and thereby have low motivation to put efforts on creative behaviors. Hence, we propose the following hypothesis:

Hypothesis 1: CCB is negatively related to employee creativity.

### The Mediating Role of Creative Self-Efficacy

Creative self-efficacy refers to “the belief one has the ability to produce creative outcomes” (Tierney and Farmer, 2002, p. 1138). Efficacy beliefs nourish intrinsic motivation (Bandura, 1986). According to the social cognitive theory, self-efficacy reflects the individuals’ beliefs in realizing the performance even under the situations that are full of uncertainty and risk (Bandura, 2001). To generate a creative outcome, it requires courage and determination because such a process is full of risks, conflicts, uncertainties, and failures (Shalley and Gilson, 2004). Bandura (1986) suggested that, when employees have a high level of self-efficacy (i.e., believing that personal efforts would lead to positive expected outcomes and avoid negative ones) and when they believe that they have knowledge and skills enabling creativity, they will feel more comfortable to accept challenges at work and to engage in creative tasks. They may even be proactive in setting challenging goals for changing the *status quo* and pursue the performance (Zhang Y. et al., 2018). Ford (1996) also pointed out that employees’ belief in their ability to complete tasks is a prerequisite for ensuring creativity at work. We therefore theorize that those employees with high CSE are more likely to welcome challenges and intrinsically motivated to finish non-standardized and non-routine tasks. They can better integrate the self-motivation and cognitive resources in producing creative outcomes. Meanwhile, employees with a high self-efficacy tend to make extra efforts (e.g., innovating the work process and methods) to achieve better performance (Gong et al., 2009). In sum, individuals with high self-efficacy have the confidence and capability to change the *status quo* and generate novel and useful ideas (Zhang Y. et al., 2018; Fang et al., 2019). As such, CSE could be a powerful precursor to creativity (Gong et al., 2009; Mittal and Dhar, 2015) and organizations consider CSE to be beneficial for promoting employee creativity and organizations’ sustainable survival and success (Huang et al., 2016). Therefore, we assume that employees’ CSE is positively related to employee creativity.

Social cognitive theory emphasizes that individual self-efficacy is a key factor in connecting external environment to individual behavior. Evidence from prior studies has reported negative associations between hindrance stressors and self-efficacy. The research by He et al. (2019b) on R&D teams in Taiwan’s technology companies contends that hindrance stressors have a destructive impact on employees’ sustained innovation behavior, and employees’ CSE plays a mediating role in this relation. When employees conduct extra-role behaviors without self-driven will, we assume that they have to adopt CCBs. Pressure is therefore created on employees to be helpful, committed in additional responsibilities, and to adopt different forms of OCB (Bolino et al., 2010). In view that an individual’s CSE decreases when he/she perceives work stress as a hindrance (He et al., 2019b), we believe that as a hindrance workplace stressor, CCB may make employees feel stressed and threatened, which will decrease employees’ internal self-efficacy of creative thinking. Specifically, first, by reducing employees’ emotional and cognitive resources, CCB could reduce employees’ confidence that is crucial for completing creative work. As for CCB, it may engender employees’ psychological strains (e.g., emotional exhaustion, depression, and tension) when they are forced to work extra hours beyond the formal workload. As such, they feel cognitively and emotionally strained by such work and feel they have little resources left, making them feel unable to perform creative work behaviors well. Second, accompanied by CCB, role conflicts and ambiguity may give employees a strong sense of helplessness and thus reduce their self-confidence. Moreover, employees who have multi extra-role activities and red tapes often lead to a lack of motivation to conduct further study, thereby hindering the development of self-efficacy. Third, CCB affects employees’ job control and decision-making. Such a feeling of insecurity will reduce employees’ cognition toward their capability. Recent research has identified abusive supervision as an important antecedent of CCB (Zhao et al., 2013). When employees are forced to engage in citizenship behaviors due to their supervisors’ abusive management, they are more likely to doubt on their own capability and thus become less confident about their creativity. Especially, for those less powerful individuals who simply cannot resist or say “no” to their supervisors or organizations, they may be more inclined to behave in a job-oriented manner than to actively engage in practicing the creative work. In other words, when confronted with CCBs, the priority for employees will therefore be task accomplishment but not necessarily being creative in the jobs. Such a priority could make them feel job security (Zhao et al., 2013; Tu et al., 2019). Accordingly, we hypothesize that:

Hypothesis 2: CSE mediates the negative relationship between CCB and employee creativity.

### The Moderating Role of Negative Affect

Workplace stressors are typically negatively related to employees’ CSE and their creative work involvement. However, employees’ responses to the stressors vary depending on the ways in which they appraise the stressors (Cavanaugh et al., 2000). In other words, how employees assess workplace stressors would have impacts on the relationship between work stressors and creativity (Byron et al., 2010). In addition, according to the interactional framework for organizational creativity (Woodman et al., 1993), creativity is the result of the interaction between personal factors and contextual factors (Zhang Y. et al., 2018). Therefore, personal factors have been studied as boundary conditions in the relationship between workplace stressors and employee creativity. In particular, scholarly attention has focused on the predictive power of personality trait measures in workplace stress management research (Ebstrup et al., 2011). Recent research noted that personality traits matter in employees’ perception of work pressure and their stress appraisal and coping processes (Carver and Connor-Smith, 2010). For example, employees with the personality trait of neuroticism tend to experience more citizenship pressure (Youn et al., 2017). Moreover, meta-analysis (Feist, 1998) and empirical studies (Herrmann and Felfe, 2014; Coelho et al., 2018) both highlighted the effects of several personality traits on employee creativity.

Among various personal factors, workplace affect (including positive affect and negative affect), which is seen as one of the main sources of self-efficacy (Bandura, 1986, 1997), has attracted considerable attention from scholars (e.g., Bang and Reio, 2016; Thundiyil et al., 2016). Employees adapt and adjust their own thinking patterns and behaviors based on their affective-motivational state. Prior studies indicate that an individual’s innovative behavior can be largely influenced by his/her affect/emotions (Amabile et al., 2005; Anderson et al., 2014). Positive affect/emotions “alter employees’ way of thinking and help them to enjoy their work” and make them “have the courage to overcome obstacles at work” (Wu and Wu, 2019, p. 3203). Such enthusiastic, energetic, and happy employees are willing to enhance their work engagement and are likely to believe that they have the ability to propose more innovative ideas. Thus, positive affect/emotions are beneficial for inspiring an individual’s innovative behavior (Tamir, 2016). In contrast, an individual’s negative affect/emotions usually lead him/her to show less engagement at work and feel depressed (Bhave and Glomb, 2016). Therefore, an individual’s negative affective/emotional state is often negatively associated with his/her work commitments and innovative behaviors (Amabile, 1988; Wu and Wu, 2019). According to the cognitive tuning model (Schwarz, 1990), negative affect, which refers to “a general factor of subjective distress, and subsumes a broad range of negative mood states, including fear, anxiety, hostility, scorn, and disgust” (Watson et al., 1988, p. 347), could shape employees’ cognitive processing (Friedman and Förster, 2010) and influence their creative outcomes (Thundiyil et al., 2016). Thus, we propose that the impacts of CCB on employees’ self-focused cognition—CSE and their creativity may also depend on the negative affect. Although the experience of conducting CCBs is usually painful, there may be significant differences for employees with different levels of negative affect.

Specifically, employees with a low negative affect are less likely to feel being emotionally overextended and exhausted in stressful conditions. As a result, they may appraise CCB less negatively or even positively by seeing it as challenging work assignments and thereby take problem-focused coping strategies. Moreover, creativity requires a substantial amount of individual resources. Previous research has shown that, positive affection will trigger an employee’s self-regulation processes “which are typically necessary to marshal the cognitive resources needed for creative solutions” (Thundiyil et al., 2016, p. 730). As such, employees’ confidence in proposing novel solutions and creative ideas will lead them to perform extra-role tasks. Individuals with low negative affect tend to be more optimistic and conduct the extra work role with more self-confidence. Therefore, we propose that the lower of negative affect that an employee has, the higher CSE and the better creative performance he/she has.

In contrast, a relatively negative affection inhibits striving for creative ideas. High negative affect could further make employees feel resource exhaustion and cognitive tension under the stressful situations. As such, they may appraise CCB as a hindrance to work assignments and thereby take emotion-focused coping strategies. Employees with a high negative affect would be more sensitive to external environmental threats and pressures, and they could tend to avoid the situation that is seen by them as problematic, threatening, and troublesome. When confronted with CCBs, these employees are likely to believe that they cannot cope with the difficult situation successfully. Therefore, we point out that employees with a high negative affect are more susceptible to CCBs, and they tend to be more pessimistic and incapable of responding to extra-role work-related stress. Furthermore, when employees experience a high negative affect, their cognitive flexibility, creative thinking, and problem-solving skills will be decreased. Generally, the high level of unpleasant affect is likely to make them feel lacking of confidence, attention, determination, and the capability to work creatively. Ultimately, he/she would develop little interest in engaging in creative work behaviors. Instead, he/she is predisposed to performance routinely, resulting in low creative performance. In sum, employees who are surrounded by negative affective states would be more prone to respond negatively to CCBs. Therefore, we expected that negative affect would amplify the disadvantageous influence of CCB on employees’ CSE.

Based on the above theoretical deduction, we suggest that negative affect is a negative factor that hinders or destroys the individual’s self-efficacy. We also tend to believe that negative affect might not serve as a direct precursor of CSE but a moderator in explaining the impacts of workplace stressors on CSE. Our study focuses on the interaction effect of CCB and negative affect on CSE. We propose:

Hypothesis 3: Negative affect moderates the negative relationship between CCB and employees’ CSE, such that the relationship will be stronger among employees with a strong negative affect as compared with employees with a weak negative affect.

As the aforementioned hypotheses indicate, CCB will indirectly influence employee creativity through CSE, and the first stage of this mediated model (i.e., the CCB–CSE association) will be moderated by the level of employees’ negative affect. We therefore expect that the indirect effect of CCB on employee creativity through CSE also will be changed by different levels of negative affect. More specifically, high CCB combined with high negative affect may lead to a more pronounced indirect destructive effect of CCB on employee creativity. By contrast, high CCB coupled with low negative affect may make the CCB–employee creativity association less salient. Thus, we propose further the moderated mediation model of the cognitive process linking CCB and employee creativity.

Hypothesis 4: Negative affect will moderate the strength of the indirect effect of CCB on employee creativity *via* CSE, such that the mediated relationship will be stronger when negative affect is high rather than low.

## Methods

### Sample and Procedure

The data were collected in 10 manufacturing firms in Southern China. With the assistance of HR managers of these firms, participants were contacted and invited to answer the survey. Participants were informed of the research purpose, the voluntary nature of participation, an assurance of anonymity and confidentiality, and the contact information of the authors (thereby reducing the social desirability bias of our study). Participants were requested to complete the questionnaires alone during working hours and return them to the authors in sealed envelopes (Richman et al., 1999). Translation and back-translation procedure (Brislin, 1980) was adopted to verify the questionnaire in Chinese.

The survey consists of two phases. In Time-1 (April 2018), 300 paper-based questionnaires were distributed. Participants were requested to evaluate the level of perceived CCB in the department and their own level of negative affect. Among the 289 returned questionnaires (a 96.33% response rate), 16 questionnaires were discarded owing to missing data. Thus, in this phase, we received 273 valid samples, representing a response rate of 91%. In Time-2 (June 2018), the survey was conducted following the same procedures. Participants were requested to assess their CSE and creativity. A coding provided by HR managers was used to match the responses received from Time-1 and Time-2. Finally, a total of 251 valid questionnaires were obtained, representing an overall response rate of 83.67%. Within the sample, 56.57% were male, 46.22% aged from 26 to 35 years, 63.35% received a university degree or higher, and 79.28% worked for their companies for more than 1 year.

### Measures

Compulsory citizenship behavior was measured through the five-item scale proposed by Vigoda-Gadot (2007). A sample item is “The management in this organization puts pressure on employees to engage in extra-role work activities beyond their formal job tasks” (from 1 = “never” to 5 = “always”). The α reliability was 0.79.

We used a three-item reflective self-rating creativity scale developed and validated by Dul et al. (2011) to assess employee creativity (i.e., the extent to which employees perceive that they produce new and potentially useful ideas), which was based on George and Zhou (2001) 13-item scale for supervisor rating of employee creativity and Noordam (2006) modification of this scale for self-rating of employee creativity. A sample item is “In my work, I often suggest new ways of performing work tasks” (from 1 = “strongly disagree” to 5 = “strongly agree”). The α reliability was 0.84. Hocevar (1981) has pointed out that “the subject, in most cases, knows more about himself than peers, supervisors, teachers, etc.” (p. 459). Similarly, as suggested by Shalley et al. (2009), employees themselves best understand what makes them creative in the workplace and hence are best suited to report creativity.

Creative self-efficacy was measured with Tierney and Farmer (2002) three-item scale. A sample item is “I have confidence in my ability to solve problems creatively” (from 1 = “strongly disagree” to 5 = “strongly agree”). The α reliability was 0.85.

Negative affect was measured by a 10-item scale from the Positive and Negative Affect Schedule (PANAS) scales (Watson et al., 1988). The scale consists of a number of words that describe different feelings and emotions, such as distressed, irritable, and nervous. Participants were requested to read each word and then indicate to what extent they have felt this way during the past few weeks (from 1 = “very slightly or not at all” to 5 = “extremely”). The α reliability was 0.84.

Control variables. Previous research on employee creativity has mainly used employee demographics, including gender, age, education, and tenure, as control variables because these variables have been found to influence employee creativity significantly (e.g., Zhou and George, 2003; Dul et al., 2011; Gu et al., 2016). Therefore, in keeping with previous research, employees’ gender, age, education, and tenure were controlled in the current study. Gender was coded: 0 = male, 1 = female. Age was coded: 1 = 25 or below, 2 = 26–35, 3 = 36–45, 4 = 46 or above. Education was coded: 1 = high school or under, 2 = vocational school, 3 = university, 4 = graduate school. Tenure was measured in months using five categories: 1 = 6 or below, 2 = 7–12, 3 = 13–24, 4 = 25–36, and 5 = 37 or above.

### Data Analytic Strategy

Because the data were self-rated and collected from the same source, we used Harman’s single-factor test as a diagnostic tool to evaluate the possible presence of a common method variance (CMV) in this study. Meanwhile, before testing the hypotheses, we also performed a series of confirmatory factor analyses (CFAs) with maximum likelihood estimation using AMOS 18.0. This is to assess how well the study variables defined their respective construct (Guarino, 2004), which helped to establish the convergent and discriminant validity of our study constructs (Bagozzi et al., 1991). Based on these, a series of hierarchical multiple regression analyses using SPSS 19.0 was performed to test CCB’s direct impact on employee creativity and the moderating role of negative affect. Further, a Sobel test and a bootstrapping analysis were performed (by using the PROCESS macro in SPSS version 19.0) to assess the statistical significance of the indirect effect of CCB on employee creativity *via* CSE. As suggested by Preacher and Hayes (2004), the Sobel test directly addresses the theme (i.e., the significance of the total effect of the independent variable on the dependent variable) reduced upon the addition of a mediator to the model, whereas the bootstrap procedure increases the power of analyses in non-experimental designs. Finally, an SPSS macro proposed by Preacher et al. (2007) and Hayes (2015) was used to test the moderated mediation hypothesis. We estimated conditional indirect effects of CCB on employee creativity through CSE at high (1 SD above the mean) and low (1 SD below the mean) negative affect. The significance of conditional indirect effects was estimated by examining the bias-corrected confidence interval (CI) obtained from bootstrapping approaches.

## Results

### Confirmatory Factor Analysis

We compared our hypothesized model (i.e., model 1, the baseline four-factor model) with two three-factor models (i.e., model 2 combining CCB and negative affect and model 3 combining CSE and employee creativity), a two-factor model (i.e., model 4 combining CCB and negative affect and combining CSE and employee creativity), and a one-factor model combining all items (i.e., model 5) (Table 1). We combined Time-1 CCB and negative affect into one factor and combined Time-2 CSE and employee creativity into one factor because previous research (e.g., Xu et al., 2015; He et al., 2019a) has generally combined constructs that were measured at the same point in time into one factor. Considering the changes in chi-square (i.e., Δχ*^2^*), three major fit indicators [i.e., comparative fit index (CFI), incremental fit index (IFI), and Tucker–Lewis index (TLI)], and root mean square error of approximation (RMSEA), our hypothesized four-factor model [with χ*^2^* of 463.86 (*df* = 183, *p* < 0.01), CFI of 0.91, IFI of 0.92, TLI of 0.89, and RMSEA of 0.08] showed better fit than other alternative models (Bentler and Bonett, 1980; Bagozzi et al., 1991). In particular, our baseline four-factor model produced a significant improvement in χ*^2^* over model 2, Δχ*^2^*(3) = 191.79, *p* < 0.01; model 3, Δχ*^2^*(3) = 100.22, *p* < 0.01; model 4, Δχ*^2^*(5) = 290.52, *p* < 0.01; and model 5, Δχ*^2^*(6) = 635.64, *p* < 0.01, suggesting a superior fit to the data than any other alternative measurement models. Therefore, the discriminant validity of the constructs was confirmed. This suggests that the participants of our survey could distinguish the focal constructs clearly. In addition, the convergent validity was also confirmed because inspection of factor loadings and factor covariance showed that all factor loadings were significant.

**TABLE 1:** Results of confirmatory factor analysis of the measurement models (*N* = 251).

Measurement Models	χ *^2^*(*df*)	Δχ *^2^*(Δ *df*)	*CFI*	*IFI*	*TLI*	*RMSEA*
Model 1: Four-factor	463.86 (183)**		0.91	0.92	0.89	0.08
Model 2: Three-factor (combined CCB and negative affect into one factor)	655.65 (186)**	191.79 (3)**	0.69	0.69	0.65	0.10
Model 3: Three-factor (combined CSE and employee creativity into one factor)	564.08 (186)**	100.22 (3)**	0.75	0.75	0.72	0.09
Model 4: Two-factor (combined CCB and negative affect into one factor, and combined CSE and employee creativity into one factor)	754.38 (188)**	290.52 (5)**	0.62	0.63	0.58	0.11
Model 5: One-factor (combined all items into one factor)	1,099.50 (189)**	635.64 (6)**	0.39	0.40	0.32	0.14

CFI, comparative fit index; IFI, incremental fit index; TLI, Tucker–Lewis index; RMSEA, root mean square error of approximation; CCB, compulsory citizenship behavior; CSE, creative self-efficacy. CCB and negative affect were measured at Time-1; CSE and employee creativity were measured at Time-2. All alternative models were compared with the four-factor model; **p < 0.01.

### Descriptive Statistics

Table 2 presents the means, standard deviations, and intercorrelations among the study variables. As shown in Table 2, employee creativity was significantly correlated with CCB (γ = –0.12, *p* < 0.05) and CSE (γ = 0.22, *p* < 0.01), and CSE was significantly correlated with CCB (γ = –0.24, *p* < 0.01). Thus, the zero-order correlations for the study variables were all in the expected direction.

**TABLE 2:** Means, standard deviations, and intercorrelations of study variables (*N* = 251).

	*M*	*SD*	1	2	3	4	5	6	7
(1) Gender	0.43	0.50	−						
(2) Age	2.27	0.99	–0.07	−					
(3) Education	2.62	0.78	–0.10	−0.12*	−				
(4) Tenure	3.37	1.14	–0.09	0.38**	–0.02	−			
(5) CCB	1.83	0.45	0.04	0.03	0.03	0.01	−		
(6) Employee creativity	3.92	0.52	0.00	0.14*	–0.04	0.25**	−0.12*	−	
(7) CSE	3.79	0.63	0.03	0.05	–0.10	0.07	−0.24**	0.22**	−
(8) Negative affect	2.41	0.43	–0.05	0.02	–0.01	0.02	–0.03	–0.07	0.01

*M*, mean; *SD*, standard deviation; CCB, compulsory citizenship behavior; CSE, creative self-efficacy. *p < 0.05 and **p < 0.01 (two-tail test).

### Hypothesis Tests

Hypothesis 1 proposed the negative effect of CCB on employee creativity (i.e., the main/direct effect). Table 3 presents the regression results. As shown by Model 2, CCB has a negative impact on employee creativity (β = –0.14, *p* < 0.05). Thus, Hypothesis 1 was verified.

**TABLE 3:** Regression summary for the mediating role of CSE and the moderating role of negative affect (*N* = 251).

	Employee Creativity		CSE		
		
	Model 1	Model 2	Model 3	Model 4	Model 5
**Control Variables**					
Gender	0.03	0.04	0.05	0.05	0.06
Age	0.02	0.03	0.02	0.02	0.01
Education	–0.02	–0.01	–0.07	–0.07	–0.05
Tenure	0.11	0.11	0.03	0.03	0.05
**Independent variable**					
CCB		−0.14*	−0.34**	−0.34**	−0.39**
**Moderate variable**					
Negative affect				0.00	0.01
**Interaction term**					
CCB × Negative affect					−0.12**
*R*^2^	0.06	0.08	0.08	0.08	0.10
*F*	4.39**	4.31**	3.95**	3.28**	4.01**
*△R^2^*	0.06	0.02	0.06	0.00	0.03
*△F*	4.39**	3.79*	15.55**	0.00	7.85**

*p < 0.05 and **p < 0.01 (two-tail test). Unstandardized regression coefficients were reported. CCB, compulsory citizenship behavior; CSE, creative self-efficacy.

Hypothesis 2 proposed the mediating effect of CSE on the CCB–employee creativity relationship. Except for the abovementioned negative relationship between CCB and employee creativity, Model 3 also shows the negative association between CCB and CSE (β = –0.34, *p* < 0.01). We then performed a Sobel test and a bootstrapping analysis based on the above regression estimates by using the PROCESS macro in SPSS version 19.0. As shown in Table 4, results of the Sobel test (effect size = –0.05, SE = 0.02, Z = –2.25, *p* < 0.05) and the bootstrapping test (point estimate = –0.05, SE = 0.02, with the 95% bias-corrected CI as –0.10 and –0.02) supported that CI did not contain zero, indicating that the indirect effect of CCB on employee creativity through CSE was statistically significant. These results lend support to our Hypothesis 2.

**TABLE 4:** Results for the indirect effect of CCB on employee creativity through CSE (*N* = 251).

Sobel for the indirect effect				Bootstrap for the indirect effect			
	
Effect size	*SE*	*Z*	*p*	Point estimate	SE	LL 95% CI	UL 95% CI
−0.05	0.02	−2.25	0.02	−0.05	0.02	−0.10	−0.02

*Bias-corrected CI is reported. Bootstrap sample size = 5,000. LL, lower limit; UL, upper limit; CI, confidence interval; CCB, compulsory citizenship behavior; CSE, creative self-efficacy.*

**FIGURE 2:**
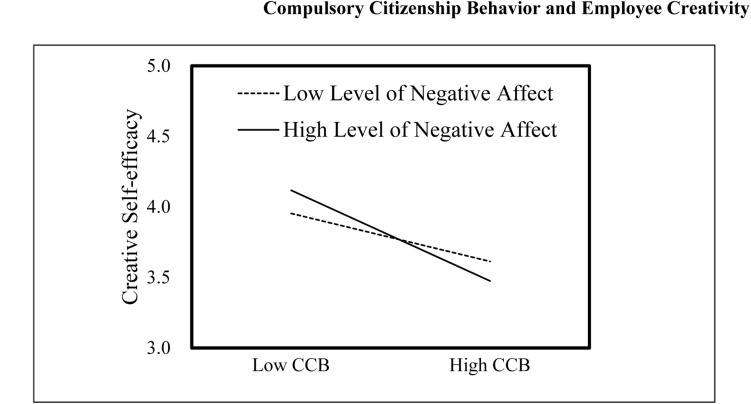
Plot of interaction between CCB and negative affect on CSE. CCB, compulsory citizenship behavior; CSE, creative self-efficacy.

We adopted hierarchical moderated regression analyses to assess the moderating effect of negative affect on the negative relationship between CCB and CSE (i.e., Hypothesis 3). We entered the control variables in Step 1, the independent variable in Step 2, the moderator in Step 3, and the interaction term in Step 4. Following Aiken and West (1991), in order to avoid multicollinearity, both the independent (CCB) and moderating (negative affect) variables were centered in the regression analyses. Consistent with our hypothesis, results shown by Model 5 in Table 3 suggested that the interaction between CCB and negative affect was negatively related to CSE (β = –0.12, *p* < 0.01) and explained an additional 3.0% of the variance in CSE, suggesting that Stage 1 of the moderation of CCB × negative affect is negative and significant. In addition, we plotted the interaction effects at different levels (i.e., +1 SD or –1 SD) of negative affect using the recommendation of Aiken and West (1991). Figure 2 shows that CCB is more negatively related to CSE when negative affect is high rather than low. Accordingly, the moderating effect of negative affect on the CCB–CSE association is as expected, and thus Hypothesis 3 is supported.

We further estimated the conditional indirect effect of CCB on employee creativity *via* CSE across levels of negative affect by bootstrapping the bias-corrected CI. The results are presented in Table 5. The indirect effect of CCB on employee creativity through CSE was stronger and significant at a high level of negative affect (effect size = –0.11, *p* < 0.01, 95% bias-corrected CI from –0.19 to –0.04) but was weaker and not significant at a low level of negative affect (effect size = –0.02, *ns*, 95% bias-corrected CI from –0.06 to 0.00). Thus, we have further evidence to support our Hypothesis 4 (the index of moderated mediation = –0.10, *p* < 0.01, 95% bias-corrected CI from –0.21 to –0.02).

**TABLE 5:** Results for conditional indirect effect of CCB on employee creativity *via* CSE across levels of negative affect (*N* = 251).

Moderator	Level	Mean	Effect size	Boot *SE*	LL 95% CI	UL 95% CI
Negative affect	Low (–1 SD)	1.99	–0.02	0.02	–0.06	0.00
	High (+ 1 SD)	2.84	–0.11	0.04	–0.19	–0.04
		Index of Moderated Mediation = –0.10		0.05	–0.21	–0.02

*Bias-corrected CI is reported. Bootstrap sample size = 5,000. Low, 1 SD below the mean; High, 1 SD above the mean. CCB, compulsory citizenship behavior; CSE, creative self-efficacy.*

## Discussion

In the current study, in response to the call for more attention to potential psychological mechanisms linking workplace stressors to employee creativity (e.g., Gu et al., 2016; Zhang Y. et al., 2018), we developed and tested the moderated mediation model on the relationship between CCB and employee creativity. The results revealed that (1) CCB is negatively associated with employee creativity; (2) the negative association between CCB and employee creativity is fully mediated by CSE; and (3) as a representative of individual differences and personality, negative affect moderates the direct effect of CCB on CSE and the mediating effect of CSE. Our findings are in line with the results of most previous studies concerning the relationship between workplace stressors (e.g., hindrance stress and abusive supervision) and employee creativity (e.g., Hon et al., 2013; Gu et al., 2016; Kwan et al., 2018; Zhang Y. et al., 2018), as well as the relationship between employees’ self-efficacy and creativity (e.g., Gong et al., 2009; Richter et al., 2012; Huang et al., 2016; Haase et al., 2018).

### Theoretical Implications

Our findings offer several contributions to the understanding of the relationship between CCB and employee creativity. First, this study deepens and expands the research on the consequences of CCB. Current research in the area mainly focuses on the concepts and the measures of CCB, the relationship and differences between CCB and OCB, and the antecedents of CCB (for example, abusive supervision and destructive leadership). The research on the impacts of CCB is underdeveloped. Recently, with the increasing attention paid to the “dark side” of OCB, some scholars (e.g., Tepper et al., 2004; Vigoda-Gadot, 2006, 2007; Zhao et al., 2014) have begun to investigate the consequences of CCB. However, these studies focus mainly on employee attitudes, psychology, and emotions, paying less attention to employee behavior and performance results. In particular, the linkage between CCB and employee creativity has not yet been examined. Therefore, by focusing on employee creativity, this study has developed a new research direction in the field of CCB, which uncovers the “dark side” research of citizenship behavior, and promotes the future research to focus more on individual-level outcomes.

Second, our findings also extend the current body of employee creativity literature by exploring its new precursors relating to workplace stressors. Research on the relationship between workplace stressors and employee creativity is attracting more and more researchers’ attention. Two main stressors have been identified in previous studies, which are challenging stressors and hindrance stressors. However, there is still a lack of in-depth research on how specific types of challenge–hindrance stressors may affect employee creativity. This study chooses CCB, a non-traditional/unconventional workplace stressor, as the research theme and examines its critical impact on employee creativity. Such a research design can extend the research focus from the traditional in-role work-related pressure (i.e., focusing on job insecurity, time pressure, and face issue) to the unnoticed extra-role work-related pressure.

Third, this work has partially uncovered the “black box” and boundary condition of the processes linking citizenship pressure to employee creativity. Most of the previous studies have acknowledged that stress can affect employee creativity, but little research has been conducted on the mediating mechanism of stress–creativity transformation (Byron et al., 2018; Zhang Y. et al., 2018). Based on the social cognitive theory, we confirmed the key intermediary role of CSE between CCB and employee creativity and further clarified the internal mechanism on how hindrance stressors could affect employee creativity. In addition, the results of this study also showed that negative affect plays a moderating role in the relationship between CCB, CSE, and employee creativity. It implies that an individual’s negative affect state can exert important influences on his/her perception of stressors. Previous studies based on the resource loss theory and social cognitive theory point out that stress can lead to the feeling of loss of individual cognitive resources, causing the aversion to physical and emotional arousal, which in turn has a negative effect on self-efficacy (Zhang Y. et al., 2018). However, our study pointed out that the loss of cognitive resources and self-efficacy caused by workplace stressors vary from one person to another because creativity is context-specific (Tierney and Farmer, 2002). We found that individuals’ personality traits related to affect/emotions can explain this phenomenon. By introducing the contingent role of individual personality traits, this study enriches the “stress–self-efficacy–creativity” relationship model based on the COR theory and social cognitive theory. To sum up, we have painted a relatively complete picture of how enforced citizenship behavior can indirectly affect employee creativity.

### Practical Implications

The results of this study provide several managerial implications on how to improve employee innovation performance through managing employee pressure, employee negative emotions, and CSE.

First, it suggests that the compulsory extra-role behaviors against employees’ will (i.e., CCB) (Vigoda-Gadot, 2007) do exist in Chinese organizations and have a negative impact on the employees’ CSE and creative behavior. Managers should be aware that citizenship behavior has double-edged effects. While it is beneficial to the improvement of organizational effectiveness, it is also costly. Citizenship behaviors are composed of both voluntary citizenship behavior and enforced citizenship behavior. When employees are forced to engage in intensive compulsory behavior, they tend to become nervous and fatigued by feeling too strong pressure and thus reduce the cognitive resources needed to generate novel ideas. At the same time, high-intensity extra-role work requirements and low working resources can also influence employees’ morale, leading them to abandon or avoid challenging aspects of their work and to apply minimum efforts in improving performance, which in turn reduces creativity. In view of this, managers should be dedicated to eliminate or reduce “bad” stressors that hinder personal development and accomplishment. They should also pay attention to the counterproductive work behaviors aimed at CCBs, especially when they manage the generation 80s and 90s. For well-behaved employees (i.e., those who hold citizenship behaviors), the organizations should prevent and compensate their resource loss through rewarding their extra-role performance accordingly. In addition, managers should guide employees to perceive citizenship pressure as a kind of challenging stressor and stimulate their intrinsic motivations to act as “good soldiers.”

Second, this study confirms the mediating role of CSE in the process of transforming CCB into decreased employee creativity. This implies that when employees cannot change the working environment, they may try to reduce the negative impact of work stress on their creativity by enhancing their CSE. Therefore, managers must attach more importance to employee CSE and provide contextual conditions or management measures that contribute to the development of employee CSE. Specifically, managers should (1) take self-efficacy into consideration during the recruitment process so that potential candidates can be identified and selected to ensure future creative work; (2) design training programs and methods to help employees become aware and improve their CSE, providing supports to employees by promoting past successful innovation experiences; and (3) improve employees’ CSE and encourage them to pursue novel, creative, and often unscripted paths of thought and action by providing more work resources and organizational supports (e.g., implicit innovation knowledge sharing, supportive leadership, trust, timely feedback, etc.) to their innovation activities.

Third, our study has identified the affect-related solutions to avoid the negative impacts of CCB on employees’ CSE and creativity. For instance, the organizations can select employees with a low negative affect in the recruitment process by paying attention to negative affect-related individual differences (i.e., distressed, upset, guilty, scared, hostile, irritable, ashamed, nervous, jittery, and afraid) (Watson et al., 1988) *via* psychological measurement or scenarios simulation and then detect and predict their affect state relying on early screening mechanisms. Another solution is to cultivate employees’ emotional intelligence. Positive affect such as happiness, satisfaction, passion, and love (Watson et al., 1988) can increase individuals’ attention resources, which will lead to the improvement of individuals’ cognitive flexibility in creative tasks, expanding their capability to think and act and then promote individual creativity (Ashby et al., 1999; Fredrickson and Branigan, 2005; Lyubomirsky et al., 2005; Hu et al., 2015). Individuals with a high emotional intelligence tend to be more optimistic and are likely to experience more positive affection. Therefore, organizations should offer workshops or courses on emotion management to employees so they can improve their emotional intelligence under citizenship pressures. In addition, managers should keep constant communication with employees and provide timely psychological consultation and guidance, eliminating or reducing the growing negative emotions among employees, and strive to create a positive emotional atmosphere.

### Limitations and Future Research

Although our work has obtained some valuable results, there are still some limitations that need to be addressed. First, although this study used a two-stage longitudinal study method, given that the measurement of the main variables used a self-rating approach, this may lead to potential social desirability and common method biases, which would influence the accuracy of the causality and external validity. Therefore, future research can consider adopting multiple-source data collection. Specifically, the evaluation of employee creativity in future research can collect data from superiors or colleagues or measure employee creativity through objective indicators such as invention patents and real innovative product quantities. At the same time, future research can also consider the combination of a scenario-based experiment and a field study to improve the validity of the findings. In addition, in order to improve the reliability and accuracy of the results, as well as examining causality between variables, in recent years, empirical research in the field of OB has shown a distinctive feature, that is, more attention is paid to the “time-effectiveness” in which the relationships between variables are examined, the time lag of each survey phase is shortened, and researchers start to adopt daily survey to collect data. Daily survey collects data through different timings in a day and lasts for a couple of weeks (e.g., from Monday to Friday, at the beginning of participants’ daily work and at the end of their workday, for a 2-week period). Due to its strength in methodology design (Brewer, 2000), daily survey has achieved high recognition by well-known journals (such as the *Academy of Management Journal*, *Journal of Applied Psychology*). Scholars in the OB field show more and more interest and attention to daily survey method (see Barnes et al., 2015; Qin et al., 2018). Therefore, future research could adopt daily investigation to test our theoretical framework.

Second, the data in this study were collected from employees in manufacturing enterprises in southern China. Therefore, whether the findings can be applicable to other regions/countries or industries (for example, service industries) requires further testing. Future research can diversify the sample source. In addition, employees’ perceptions of and reactions to CCB is culture-specific. For example, in organizations that emphasize collectivism, self-sacrifice, and ethics, employees with high pro-social motivation, responsibility, and altruism may feel that engaging in OCBs is a normal thing. Therefore, their CSE and creativity may be affected by CCB to a lesser extent. In view of this, in-depth comparative study of CSE and creativity under CCB between Chinese and employees of other cultural backgrounds calls for more attention.

Third, our focus on CSE as a mediator and negative affect as a moderator has only partially revealed the underlying processes linking CCB to employee creativity and the boundary conditions. For the mediating mechanism, future research can use job demands–resource model to construct the emotional/affective paths of the CCB–creativity link and investigate the potential mediating role of emotional exhaustion, citizenship fatigue, and work–family/leisure conflict. Furthermore, based on the social cognitive theory, future research can construct other cognitive paths for converting CCB into decreased creativity such as examining the potential mediating role of psychological contract violation, psychological safety, moral disengagement, and moral identity. Regarding the moderating mechanism, future research can analyze personality traits (e.g., Big Five, creative personality), affective commitment, organizational identification, perceived organizational support, leader–member exchange/supervisor–subordinate *guanxi*, team–member exchange, ethical leadership, organizational context (such as team innovation atmosphere, organizational innovation culture), which can further provide a more comprehensive theoretical explanation to the relationship between CCB and employee creativity.

Fourth, our findings suggest that there is a significant negative correlation between CCB and employee creativity. However, some scholars believe that although excessive work stress will reduce the time for employees to be creative, the appropriate level of work pressure can boost employees’ intrinsic motivation and thus enhance employee creativity (e.g., Amabile et al., 1996; Zhou and Long, 2011). For example, Antwi et al. (2019) have interrogated the job demand stressors (including challenge and hindrance stressors)–creativity curvilinear relationships. Prior research also indicated that the impact of citizenship pressure is twofold: harmful (see for example Bolino et al., 2010) and beneficial (see for example Cates et al., 2010). Therefore, the relationship between citizenship pressure and employee creativity may not be a simple linear relationship. It may be a curvilinear relationship (for example, the reversed U-shape relationship), and perhaps a moderate level of citizenship pressure can stimulate maximum employee creativity. In view of this, it is worthwhile to explore how employees’ creativity will change with the intensity of CCB over a long period of time. In other words, future research can further test the potential double-edged sword effect of CCB.

## Conclusion

Citizenship behavior is an individual extra-role work behavior that can improve organizational effectiveness. In order to maintain firms’ competitive advantage, more and more Chinese companies use various means to force employees to engage in more citizenship behaviors besides their assigned job duties. However, employees’ involuntary engagement in citizenship behaviors may charge employees’ additional working and cognitive resources, and the loss of such resources is likely to destroy employees’ CSE and creativity. Therefore, based on the social cognitive theory and COR theory, we proposed a moderated mediation model for examining how CCB interacts with negative affect to influence employees’ CSE and their subsequent creative performance. The results suggest that CCB has significant destructive effects on the organizations in terms of decreasing the creativeness level of employees, and CSE can serve as a mediator in this relationship, especially when employees’ negative affect is high rather than low. All in all, the current study has provided some new paths (i.e., self-efficacy-related cognitive process and individual characteristics perspectives) to view the workplace stressors–employee creativity relation.

## Data Availability Statement

The datasets generated for this study are available on request to the corresponding author.

## Ethics Statement

An ethics approval was not required as per institutional guidelines and national laws and regulations because no unethical behaviors existed in this study. We just conducted paper-pencil test and were exempt from further ethics board approval since our study did not involve human clinical trials or animal experiments. In the survey process, all participants were informed that participation was voluntary and assured that their responses would be only used for our study and kept confidential strictly. Therefore, only those who were willing to participate were recruited. To ensure confidentiality, the questionnaires completed during their working hours were directly returned to the first author in sealed envelopes.

## Author Contributions

PH, HZ, and YW designed and supervised the study. PH collected the data. PH and HZ analyzed the data. PH, QZ, and CJ wrote the manuscript. All authors contributed equally to this manuscript, and reviewed and approved this manuscript for publication.

## Conflict of Interest

The authors declare that the research was conducted in the absence of any commercial or financial relationships that could be construed as a potential conflict of interest.
